# Serum zinc-α2-glycoprotein levels are elevated and correlated with thyroid hormone in newly diagnosed hyperthyroidism

**DOI:** 10.1186/s12902-019-0336-9

**Published:** 2019-01-22

**Authors:** Xin-Hua Xiao, Xiao-Yan Qi, Jiao-Yang Li, Yi-Bing Wang, Ya-Di Wang, Zhe-Zhen Liao, Jing Yang, Li Ran, Ge-Bo Wen, Jiang-Hua Liu

**Affiliations:** Department of Metabolism and Endocrinology, The First Affiliated Hospital of University of South China, Hengyang, 421001 China

**Keywords:** Hyperthyroidism, Zinc-α2-glycoprotein, Adipokine, Thyroid hormone

## Abstract

**Background:**

Zinc-α2-glycoprotein (ZAG) is a recently novel lipolytic adipokine implicated in regulation of glucose and lipid metabolism in many metabolic disorders. In vitro and animal studies suggest that thyroid hormones (TH) up-regulates ZAG production in hepatocytes. However, there is no data evaluating the possible relationship between ZAG and TH in a human model of hyperthyroidism. The objective of the present study is to assess the association of serum ZAG levels with TH and lipid profile in patients with hyperthyroidism before and after methimazole treatment.

**Methods:**

A total of 120 newly diagnosed overt hyperthyroidism and 122 healthy control subjects were recruited. Of them, 39 hyperthyroidism patients were assigned to receive methimazole treatment as follow-up study for 2 months.

**Results:**

The clinical consequence showed that serum ZAG levels were elevated in patients with hyperthyroidism (*P* < 0.01). Adjust for age, gender and BMI, serum ZAG levels were positively related with serum free T3 (FT3), free T4 (FT4) levels and negatively correlated with serum total cholesterol (TC), low density lipoprotein cholesterol (LDLC) levels in hyperthyroidism subjects (all *P* < 0.01). After methimazole treatment, serum ZAG levels were decreased and the decline was associated with decreased FT3, FT4 and increased TC levels (all *P* < 0.001).

**Conclusion:**

We conclude that ZAG may be involved in the pathogenesis of lipid metabolism disorder in patients with hyperthyroidism.

**Trial registration:**

ChiCTR-ROC-17012943. Registered 11 October 2017, retrospectively registered.

**Electronic supplementary material:**

The online version of this article (10.1186/s12902-019-0336-9) contains supplementary material, which is available to authorized users.

## Introduction

Hyperthyroidism is a clinical situation where there is excess TH in the circulation due to increased synthesis of hormone from a hyperactive thyroid gland [1]. In addition to typical clinical symptoms like resting energy expenditure and weight loss directly related to excess TH, the patients with hyperthyroidism are likely to accompanied by changes in lipid metabolism [2, 3]. However, the underlying mechanisms are not fully elucidated.

Adipose tissue secretes a variety of active biological substances called adipokines that act in an autocrine, paracrine and endocrine manner [4]. Several lines of evidence have shown that adipokines, such as adiponectin, leptin, resistin and fibroblast growth factor 21, etc., play an important role in regulating energy expenditure and metabolism of lipids [5–8]. Recently, researchers demonstrate that apart from abnormal circulating levels of TH and thyroid-stimulating hormone (TSH), changes in profile of adipokines (like adiponectin, leptin and resistin, etc.) also have been found in patients with hyperthyroidism [9–11]. Moreover, adipocytes express high levels of TH and TSH receptors which function similar to those in thyroid, suggesting TH may participates in the regulation of adipocyte functions [12]. Thereby, thyroid dysfunction may influence secretion of adipokines, which contributes to lipids metabolic disorders.

Zinc-alpha-2-glycoprotein (ZAG) is a recently characterized adipokine synthesized and secreted mainly by adipose tissues and liver [13]. It is a 43-kDa soluble glycoprotein first isolated from human plasma and proposed as a tumour-derived cancer cachexia [14]. It is found in various bodily fluids such as plasma, semen, sweat, milk and cerebrospinal fluid [15]. The plasma concentration of ZAG is affected by several factors, including body weight and health status. ZAG has been found to have a wide range of biological activities, but the recent interest in ZAG function comes from its specific lipolytic action and its potential role in body weight regulation. Very recently, we further explained the functions of ZAG, it can protect against obesity-associated fatty liver by ameliorating hepatic steatosis, insulin resistance and inflammation, as well as promote browning in adipocytes, once again indicating its novel role in lipid metabolism [16–18].

Given that both TH and ZAG are involved in regulating energy expenditure and metabolism of lipids, moreover, in vitro and animal studies suggest that TH up-regulates ZAG production in hepatocytes [19], we suspect that overt hyperthyroidism might alter the production of ZAG. However, so far, there are limited human studies of ZAG expression and little is known of ZAG’s role in hyperthyroidism. In this study, we investigated the association of serum ZAG levels with TH and lipid profile in patients with hyperthyroidism before and after methimazole treatment.

## Materials and methods

### Subjects

A total of 120 consecutive newly diagnosed overt hyperthyroidism (37 men and 83 women) were enrolled from the First Affiliated Hospital of University of South China (Hengyang, China), from October 2015 to August 2016. All of the subjects were diagnosed with overt hyperthyroidism by typical symptom, elevated serum TH, reduced thyroid-stimulating hormone (TSH), and TSH receptor antibody (TRAb) levels in these patients might increase or normal. Additionally, 122 healthy individuals who had undergone a routine physical examination were recruited as the control group. All of the hyperthyroidism patients were drug-naive before recruitment. Thirty-nine hyperthyroid patients received methimazole treatment for two months. No dietary recommendations were given. Exclusion criteria for both groups included age < 18 years, BMI > 35 kg/m^2^, known cardiovascular disease, neoplasms, smoking, diabetes, hypertension, and renal impairment (serum creatinine 120 μmol/L).

### Ethics statement

The study protocol was approved by the Ethics Committee of the First Affiliated Hospital of University of South China (Number: 2015-05-01), and written informed consent was obtained from all participants before their inclusion in the study. The items of the consent form include aim, inclusion and exclusion criteria, procedures, harm and benefit, medical care, privacy and right, and withdrawal. All procedures were in accordance with the Helsinki Declaration.

### Biochemical measurements

A standard questionnaire was used to collect the information about health status and medications. Blood samples were collected from 8 to 9 am after a 12-h overnight fast, and serum was separated and stored at − 20 °C for assay. Fasting plasma glucose (FPG), FT3, FT4 and TSH were measured electrochemiluminescence immunoassay (Roche Diagnostics). Total cholesterol (TC), triglyceride (TG), low density lipoprotein cholesterol (LDLC), and high-density lipoprotein cholesterol (HDLC), alanine aminotransferase (ALT), aspartate aminotransferase (AST), total bilirubin (TBIL) and conjugated bilirubin (DBIL) levels were measured by colorimetric enzymatic assays with an autoanalyzer. The serum concentrations of ZAG were determined using ELISA according to the manufacturer’s protocols (Biovendor, Modrice, Czech Republic).

### Statistical analysis

All analyses were performed with Statistical Package for Social Sciences version 17.0 (SPSS, Chicago, IL, USA). Normal distributed data were expressed as mean ± SD. Data that were not normally distributed, as determined using Kolmogorox-Smirnov test, were logarithmically transformed before analysis and expressed as median with interquartile range (IQR). *χ*^2^ and one-way ANOVA tests were used for comparison of categorical and continuous variables, respectively. The Student’s paired *t* test was used for comparison of the data before and after anti-thyroid treatment. The Pearson correlation coefficient was used for analyses of correlations. Multiple stepwise regression was performed to determine variables that had independent associations with serum ZAG. *P-*values < 0.05 (two-sided) were considered statistically significant.

## Results

### Subject characteristics

The clinical characteristics of study subjects are shown in Table 1. The age and sex are comparable between controls and patients. Compared to controls, patients with hyperthyroidism had higher FT4, FT3, TRAb, ZAG levels and lower TSH levels (all *P* < 0.001). In addition, DBP, ALT, TBIL and DBIL levels were increased and BMI, WC, TC, TG, LDLC levels were decreased in hyperthyroid patients (*P* < 0.01). However, no significant difference was found in SBP, FPG, HDLC and AST levels between two groups.

**Table 1:** Clinical and biochemical features in study subjects

Variables	Controls	Hyperthyroidism patients	*P* value
No. of subjects	122	120	–
Gender, M/F	40/82	35/85	0.580
Age (years)	41.89 ± 9.64	40.22 ± 10.88	0.208
BMI (kg/m^2^)	23.60 ± 2.24	21.24 ± 2.91	< 0.001
WC (cm)	81 ± 8	76 ± 7	< 0.001
SBP (mmHg)	119 ± 11	121 ± 10	0.174
DBP (mmHg)	73 ± 7	77 ± 8	< 0.001
TC (mM)	4.76 ± 1.00	3.79 ± 0.85	< 0.001
TG (mM)^£^	1.38 (0.99,1.91)	1.07 (0.86,1.37)	< 0.001
LDLC (mM)	2.78 ± 0.83	1.62 ± 0.55	< 0.001
HDLC (mM)	1.46 ± 0.36	1.53 ± 0.40	0.194
ALT (IU/L)^£^	21 (14,29)	25 (17,38)	< 0.001
AST (IU/L)^£^	23 (18,29)	24 (19,31)	0.081
TBIL (μmol/l)^£^	11.4 (8.2,15.3)	13.6 (10.3,18.2)	0.002
DBIL (μmol/l)^£^	3.3 (2.5,4.5)	4.6 (3.4,6.2)	< 0.001
FPG (mM)	5.13 ± 0.54	5.21 ± 0.61	0.293
FT3 (pmol/L)£	4.64 (4.01,5.63)	13.72 (9.71,19.18)	< 0.001
FT4 (pmol/L)£	16.36 (14.30,18.43)	39.00 (29.46,54.32)	< 0.001
TSH (uIU/ml)	2.560 (1.558,3.390)	0.008 (0.004,0.049)	< 0.001
TRAb (IU/ml)^£^	3.10 (0.70,5.58)	29.88 (20.66,63.68)	< 0.001
ZAG (mg/L)	47.81 ± 12.90	66.51 ± 13.53	< 0.001

### Correlations of serum ZAG levels with thyroid hormone and other clinical parameters

**Fig. 1:**
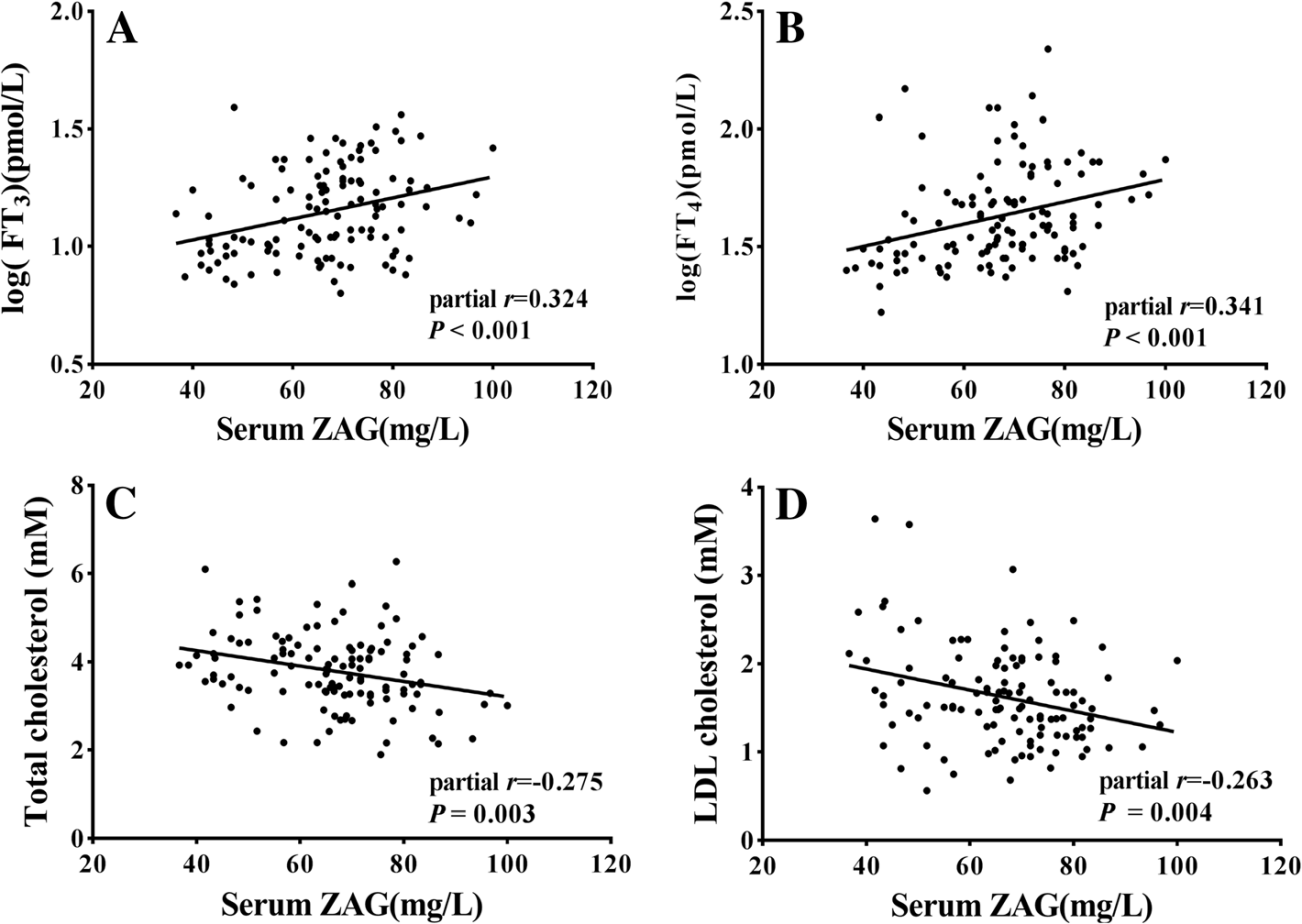
Correlations of serum levels of ZAG with (**a**) FT3 (log transformed), (**b**) FT4 (log transformed), (**c**) TC and (**d**) LDLC in 120 hyperthyroidism subjects. The *r* was assessed by partial correlation analysis with adjusted age, gender and BMI

We also investigated the associations between ZAG and the other parameters in hyperthyroidism subjects. ZAG was negatively correlated with BMI, TC, LDLC and TSH (all *P* < 0.05), positively correlated with AST, FT3, FT4 (all *P* < 0.05). After adjustment for age, gender and BMI, serum ZAG levels were positively related with serum FT3, FT4 levels, the correlation coefficient was 0.324 (*P* < 0.001) and 0.341 (*P* < 0.001), respectively (Fig. 1 a-b). In addition, serum ZAG levels were negatively correlated with serum TC and LDLC levels (correlation coefficient = − 0.275 and − 0.263, all *P* < 0.01, Fig. 1 c-d). However, there was no significant correlation between serum ZAG and SBP or DBP, TG, HDLC, ALT, TBIL, DBIL, TSH, TRAb. To determine which parameters were independently associated with serum ZAG, multiple stepwise regression analysis was performed. Age, gender, BMI, TC, LDLC, ALT, AST, FT3, FT4, and TSH were analyzed. FT3 was found to be independently associated with serum ZAG (*β* = 1.230, *P* < 0.001).

### Influences of methimazole treatment on metabolic parameters and serum ZAG levels

**Fig. 2:**
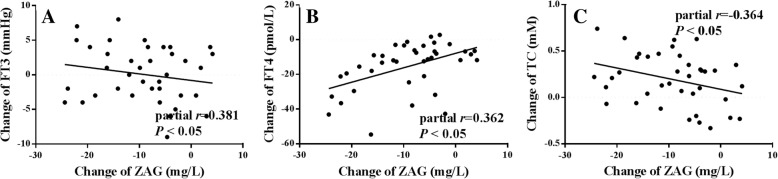
After methimazole treatment, the change of serum ZAG levels was associated with change of FT3, FT4 and TC levels in 39 hyperthyroidism patients

No serious adverse events were recorded during the follow-up observation study. The levels of FT3 and FT4 were significantly decreased (*P* < 0.01) from baseline while the levels of Weight, TC and LDLC were dramatically increased (*P* < 0.01) in patients with overt hyperthyroidism (Table 2). Serum ZAG levels was decreased from 64.85 ± 12.84 mg/l to 55.72 ± 8.83 mg/l after methimazole treatment (*P* < 0.001) (Table 2). Interestingly, the decreased ZAG levels were significantly correlated with the decreased FT3, FT4 and increased TC levels, even after adjustment for the independent variables of age, gender and BMI (FT3: partial *r* = 0.381; FT4: partial *r* = 0.362, TC: partial *r* = − 0.364, all *P* < 0.05) (Fig. 2).

**Table 2:** Comparison of clinical parameters after methimazole treatment in patients with overt hyperthyroidism

Parameters	Clinical hyperthyroidism group (*n* = 39)	
	Baseline	After treatment
Age, y	40.31 ± 11.45	40.31 ± 11.45
Gender, M/F, n	15/24	15/24
Weight (kg)	54.60 ± 6.72	56.83 ± 6.38^**^
BMI, kg/m^2^	20.67 ± 2.75	21.90 ± 2.54
SBP (mmHg)	120 ± 9	118 ± 10
DBP (mmHg)	76 ± 7	74 ± 7
TC (mM)	3.63 ± 0.83	3.82 ± 0.76^**^
TG (mM)	1.10 ± 0.37	1.15 ± 0.34
LDLC (mM)	1.51 ± 0.46	1.81 ± 0.51^**^
HDLC (mM)	1.48 ± 0.42	1.49 ± 0.35
ALT (IU/L)	31 ± 21	32 ± 15
AST (IU/L)	30 ± 11	31 ± 12
FT3 (pmol/L)	14.68 ± 6.39	9.40 ± 4.59^**^
FT4 (pmol/L)^£^	34.12 (27.61,48.93)	25.69 (21.05,30.68)^**^
TSH (pmol/L)^£^	0.006 (0.004,0.010)	0.180 (0.094,0.400)^**^
ZAG (mg/L)	64.85 ± 12.84	55.72 ± 8.83^**^

## Discussion

In this study, we found that serum ZAG levels were increased in patients with newly diagnosed hyperthyroidism and declined after methimazole treatment. Serum ZAG levels were positively related with serum FT3, FT4 levels and negatively correlated with serum TC and LDLC levels in hyperthyroidism subjects after adjustment for age, gender and BMI. Moreover, the decline of ZAG levels was significantly correlated with the decrease of FT3, FT4 and increased TC levels. These findings present for the first time the clinical relevance between TH and serum ZAG levels in hyperthyroidism subjects.

Epidemiological studies have shown that hyperthyroidism results in a hyper-metabolic state associated with increased energy expenditure causing weight loss [20]. Significantly, the patients with hyperthyroidism are likely to accompanied by changes in lipid metabolism [2]. Our study was consistent with the notion and showed that hyperthyroid patients had decreased levels of blood lipids and displayed an ectomorphic type as measured by BMI and WC. Numerous putative underlying mechanisms have been proposed to explain this changes in lipid metabolism: i) TH can directly trigger a series of pathway mainly involved in lipid metabolism and energy homeostasis, such as PI3K/Akt, MAPK/ERK, SIRT1, peroxisome proliferator activated receptors (PPARs), etc. [21, 22]. ii) More importantly, TH participates in the regulation of adipocyte functions including secreting adipokines [10, 23, 24]. As refered before, there is striking evidence that TH excess lead to prominent changes in classical adipokines (like adiponectin, leptin and resistin, etc.), we wonder serum ZAG, a novel lipid-mobilizing adipokine, whether changed in hyperthyroidism patients.

To the best of our knowledge, there is only one study investigating serum ZAG concentration in hyperthyroid patients [19]. It concluded that serum ZAG levels were increased in patients with hyperthyroidism and declined after methimazole treatment. However, they did not have data demonstrating the association of serum ZAG levels with TH and lipid profile in patients with hyperthyroidism before and after treatment. Here, we detected consistent changes in serum ZAG levels in patients with newly diagnosed hyperthyroidism. Furthermore, association analyses showed that serum ZAG levels were positively related with serum FT3, FT4 levels after adjusting for age, gender and BMI in patients with newly diagnosed hyperthyroidism both before and after treatment with methimazole. Remarkably, multiple regression confirmed the FT3 was independently related to serum ZAG. Combined with a previous in vivo study which suggested TH could increase ZAG production [19], we therefore concluded that the increased circulating ZAG levels in hyperthyroidism patients may partly due to TH excess.

Many works documented the alteration in serum concentrations of ZAG was closely linked with dyslipidemia in various endocrine metabolic disorders, such as type 2 diabetes mellitus, polycystic ovary syndrome, growth hormone deficiency, Cushing’s syndrome, obesity, non-alcoholic fatty liver disease and metabolic syndrome [25–31]. In addition, Studies performed by Olofsson et al. showed that serum levels of ZAG were significantly correlated with serum TC and TG levels, and a polymorphism in the ZAG gene was also associated with circulating TC levels in healthy and obese Swedish population, suggesting that ZAG is involved in lipid metabolism [32]. In good agreement, we observed in the present study that the serum levels of ZAG were negatively associated with TC, LDLC levels after adjusting for age, gender and BMI in patients with newly diagnosed hyperthyroidism both before and after treatment with methimazole. Hence, these lines of clinical evidences suggest a potential role of ZAG in pathogenesis of metabolic syndrome.

There are several limitations in our current study. First, the study was a non-randomized controlled trial, which may cause some bias. Second, the sample size was relatively small and consisted entirely of Chinese people, which may have hampered the generalization of our findings. Despite there are some limitations, it seems likely that ZAG may have a pathophysiological role in lipid metabolism in patients with hyperthyroidism. Further detailed studies are still needed to better elucidate the underlying molecular mechanisms.

## Conclusion

Our study demonstrates that serum ZAG levels are elevated in patients with hyperthyroidism and decreased after methimazole treatment. The decline of ZAG was associated with FT3, FT4 and TC levels. Our findings provide clinical evidence that ZAG may be involved in the pathogenesis of lipid metabolism disorder in patients with hyperthyroidism (Additional file 1).

## Additional file

Additional file 1: Baseline and follow-up data of study subjects. Baseline data included anthropometric parameters and biochemical parameters in 122 control subjects and 120 patients with newly diagnosed hyperthyroidism. Follow-up data contained anthropometric parameters and biochemical parameters of 39 cases overt hypothyroid patients before and after methimazole treatment. (XLS 64 kb)

## Abbreviations

ALT: Alanine aminotransferase AST: Aspartate aminotransferase
BMI: Body mass index DBP: Diastolic blood pressure
DBIL: Conjugated bilirubin FPG: Fasting plasma glucose
FT3: Free T3 FT4: Free T4 HDLC: High-density lipoprotein cholesterol
LDLC: Low-density lipoprotein cholesterol SBP:Systolic blood pressure
TBIL: Tobal bilibrubin TC: Total cholesterol TG: Triglyceride
TH: Thyroid hormone TRAb: TSH receptor antibody
TSH: Thyroid-stimulating hormone WC: Waist circumference
ZAG: Zinc-α2-glycoprotein
